# Synthesis and characterization of NIR-responsive Au_rod_@pNIPAAm-PEGMA nanogels as vehicles for delivery of photodynamic therapy agents

**DOI:** 10.1186/1556-276X-8-4

**Published:** 2013-01-02

**Authors:** Ting Shang, Cai-ding Wang, Lei Ren, Xin-hua Tian, Dong-hui Li, Xue-bin Ke, Min Chen, An-qi Yang

**Affiliations:** 1 Department of Biomaterials, Research Center of Biomedical Engineering, College of Materials, Xiamen University, Xiamen 361005, China; 2 State Key Laboratory for Physical Chemistry of Solid Surfaces, Xiamen University, Xiamen 361005, China; 3 Neurosurgical Department of Affiliated Zhongshan Hospital, Xiamen University, Xiamen 361004, China; 4 College of Medicine, Xiamen University, Xiamen 361005, China

**Keywords:** NIR-responsive, Au_rod_@pNIPAAm-PEGMA nanogel, LCST, singlet oxygen, PDT

## Abstract

A near-infrared (NIR)-responsive Au_rod_@pNIPAAm-PEGMA nanogel was synthesized in two steps, growing a PEGMA monolayer on the surface of gold nanorods (AuNRs), followed by *in situ* polymerization and cross-linking of *N*-iso-propylacrylamide (NIPAAm) and poly-(ethylene glycol)-methacrylate (PEGMA). The AuNRs and Au_rod_@pNIPAAm-PEGMA nanogel were characterized by UV–vis spectroscopy, Raman spectroscopy, Fourier transform infrared spectroscopy, and transmission electron microscopy, respectively. The lower critical solution temperature of the Au_rod_@pNIPAAm-PEGMA nanogel could be tuned by changing the molar ratio of NIPAAm/PEGMA. The NIR-mediated drug release behavior of the Au_rod_@pNIPAAm-PEGMA nanogel was studied with zinc phthalocyanines (ZnPc_4_) as a drug model. It was also demonstrated that the loaded ZnPc_4_ could keep the capability of generating singlet oxygen, and the *in vitro* study showed a great photodynamic therapy (PDT) effect on Hela cells. It thus indicated the potential of this Au_rod_@pNIPAAm-PEGMA nanogel for application as a drug carrier in PDT, which might make contributions to oncotherapy.

## Background

Gold nanoparticles including nanoshells, nanocages, and nanorods have drawn increasing attention in photodynamic therapy (PDT), drug delivery, and diagnostic imaging field in recent years [1-5]. Among them, gold nanorods (AuNRs) are of particular interest due to their unique optical properties. With the different aspect ratios and the resulting longitudinal surface plasmon resonance (SPR), AuNRs exhibit an absorption band in the near-infrared (NIR) region [6], which conduces to higher photothermal conversion and also shows significant biomedical application in view of the penetration of NIR light into biological tissues [7,8].

Poly(*N*-isopropylacrylamide) (pNIPAAm) gel, as one of the most widely studied temperature-responsive polymers [9-11], undergoes phase transition in water when the temperature increases or decreases beyond its lower critical solution temperature (LCST; approximately 32°C) [12,13]. Besides, its LCST can be tuned by the addition of a comonomer during polymerization [14,15]. Combining this temperature-responsive gel and gold nanoparticles together, the temperature-responsive gel can be induced to collapse by the photothermal conversion of gold nanoparticles, which gives rise to much possibility for the application of the temperature-responsive gel in drug delivery. The composites of Au@pNIPAAm have been synthesized and studied in many works [16-18]. However, the combination mostly through the physical embedding effect or electrostatic interaction between gold nanoparticles and pNIPAAm may make the composites lack long-term stability, especially in the biological environment. Our previous work has reported the synthesis of a core-shell structured multifunctional hybrid Au@IPN-pNIPAAm nanogel in which the hydrogel could be chemically grafted onto a single gold nanoparticle [19].

Herein, we developed a new way to immobilize pNIPAAm combined with poly-(ethylene glycol)-methacrylate (PEGMA) on the surface of AuNRs through chemical grafting to obtain NIR-responsive Au_rod_@pNIPAAm-PEGMA nanogel. ZnPc_4,_ a photosensitizer, was used as drug model to investigate the drug loading and release properties of the Au_rod_@pNIPAAm-PEGMA nanogel. The capacity of generating singlet oxygen of ZnPc4 after being loaded in the Au_rod_@pNIPAAm-PEGMA nanogel was measured, and the *in vitro* PDT was also studied. Our current results suggested the potential of Au_rod_@pNIPAAm-PEGMA nanogel as a carrier in PDT.

## Methods

### Synthesis of PEGMA-SH compound

Concentrations of 1.0 mmol 5,5^′^-dithiobis (2-nitrobenzoic acid) (DTNB) and 2.0 mmol dicyclohexylcarbodiimide (DCC) were dissolved in 50 mL of dichlormethane, followed by the addition of 2.2 mmol 4-dimethylaminopyridine (DMAP) and 2.0 mmol PEGMA. The mixture was degassed with nitrogen and then stirred for 48 h at room temperature. After filtration, the filtrate was washed sequentially with water, 5% acetic acid, and water. Then, the organic phase was dried over magnesium sulfate, filtered, and evaporated to dryness. The product was dissolved in 100 mL of water/ethanol (*V*/*V*, 4/1) with the addition of 2 mL of 1 M sodium borohydride (NaBH_4_) and stirred for 2 h, and was used without further purification.

### Synthesis of Au_rod_@pNIPAAm-PEGMA nanogel

AuNRs with a length of 50 nm were synthesized using the seed-mediated growth method as reported previously [20]. Subsequently, 0.1 mmol PEGMA-SH was added to 25 mL of the as-prepared AuNRs suspension (1.6 × 10^−6^ μmol) and continuously stirred for 5 h at room temperature. Au_rod_@PEGMA was collected by centrifugation at 9,500 rpm for 12 min and then re-dispersed in 15 mL of the deionized water, followed by the addition of 1.8 mmol NIPAAm, 0.2 mmol PEGMA, 86.69 μmol sodium dodecyl sulfate (SDS), and 12.97 μmol *N*,*N*-methylenebisacrylamide (BIS). The mixture was heated to 75°C with stirring and maintained in vacuum. After equilibration for 1 h, the polymerization was initiated by adding 109.6 μmol ammonium persulfate (APS). The reaction was allowed to proceed for 4 h at 75°C and terminated by opening the system to air. The resulting Au_rod_@pNIPAAm-PEGMA nanogels were purified by repeated centrifugation (9,000 rpm for 12 min) and subsequently lyophilized for further use.

### Characterization

The optical properties of AuNRs and Au_rod_@pNIPAAm-PEGMA nanogels were characterized by an UV–vis spectrophotometer (DUTM800, Beckman Coulter, Brea, CA, USA) with a scanning speed of 1,200 nm/min from 400 to 1,000 nm. The transmission electron microscopy (TEM) images were obtained from a JEM 2100 microscope (JEOL Ltd., Tokyo, Japan) operating at an acceleration voltage of 200 kV. Raman spectra were performed on an UV-1000x instrument (Renishaw, Wotton-under-Edge, UK) (path length = 200 nm) using a red light-emitting diode laser (*λ* = 785 nm, 0.5 mW). A Fourier transform interferometer (AVATAR360, Nicolet Instrument Corporation, Madison, WI, USA) was used to record the absorption spectra of AuNRs and Au_rod_@pNIPAAm-PEGMA nanogels between 400 and 4,000 cm^−1^ at a spectral resolution of 4 cm^−1^.

### LCST measurement of Au_rod_@pNIPAAm-PEGMA nanogel

In order to investigate the thermal property of the Au_rod_@pNIPAAm-PEGMA nanogel, nanogels with different molar ratios of NIPAAm/PEGMA (1:0, 18:1, 12:1, 9:1, 6:1, 4.5:1) were synthesized. LCSTs of nanogels were measured through turbidimetric measurement. The concentration for each Au_rod_@pNIPAAm-PEGMA nanogel in the deionized water was maintained at 1 mg/mL. The light transmittances at 600 nm were then measured by an UV–vis spectrophotometer (TU-1901, Beijing Purkinje General Instrument Co. Ltd, Beijing, China) equipped with a temperature-controlled sample holder, and the heating rate was set at 0.1°C/min. The LCST was defined as the initial break point in the resulting transmittance versus temperature curves.

### ZnPc_4_ loading and NIR-mediated ZnPc_4_ release

Two milligrams of Au_rod_@pNIPAAm-PEGMA nanogels and 2 mg of ZnPc_4_ were dispersed in 10 mL of *N*,*N*-dimethyl formamide (DMF) and stirred for 24 h at room temperature. The ZnPc_4_-loaded Au_rod_@pNIPAAm-PEGMA nanogels were then collected by centrifugation (9,000 rpm for 12 min). To determine the amount of unloaded ZnPc_4_, the supernatant was analyzed by an UV–vis spectrophotometer (DUTM800, Beckman Coulter) at 680 nm where ZnPc_4_ has a maximum absorption. The loading efficiency was calculated according to the following formula:

$$\text{Loading}\;\text{efficiency}=\frac{W_{t}-W_{0}}{W_{t}}\;\times 100\%,$$

where *W*_t_ represents the total amount of ZnPc_4_ and *W*_0_ represents the unloaded amount of ZnPc_4_.

For the NIR-mediated ZnPc_4_ release, 5 mL of the ZnPc_4_-loaded Au_rod_@pNIPAAm-PEGMA nanogel suspension (1 mg/mL) was placed into dialysis bags (molecular weight cutoff, 8 to 14 kDa) and irradiated by an 808-nm laser (0 to 400 mW/cm^2^) for different times (0 to 60 min). To determine the amount of ZnPc_4_ released, the dialysate was removed and subsequently analyzed by an UV–vis spectrophotometer (DUTM800, Beckman Coulter). The release efficiency was calculated as follows:

$$\;\text{Release}\;\text{efficiency}=\frac{W_{r}}{W_{l}}\times 100\%,$$

where *W*_r_ represents the released amount of ZnPc_4_ and *W*_l_ represents the loaded amount of ZnPc_4_.

### Singlet oxygen detection

The generation of singlet oxygen (^1^O_2_) from ZnPc_4_ loaded in the Au_rod_@pNIPAAm-PEGMA nanogel was determined by the transformation of 9,10-dimethylanthracene (DMA) which exhibits a maximum absorption at 377 nm [21]. The DMA can react irreversibly with ^1^O_2_ to yield an endoperoxide. The reaction could be monitored by recording the decrease in the absorption at 377 nm. In a typical experiment, 0.105 mg of the Au_rod_@pNIPAAm-PEGMA nanogel loaded with 0.0135 μmol ZnPc_4_ was dispersed in 3 mL of DMF, and then, 0.45 μmol DMA was added. Pure ZnPc_4_ (0.0135 μmol) was used as a control. The solutions were then irradiated with a LED lamp (680 nm, 10 mW/cm^2^) or a NIR laser (808 nm, 400 mW/cm^2^). The absorption measurements followed by irradiation were carried out every 5 min.

### Light-induced *in vitro* PDT effect

Hela cells were seeded into 24-well cell culture plates (1 × 10^5^ cells/well) and incubated for 24 h. After being treated with ZnPc_4_-loaded Au_rod_@pNIPAAm-PEGMA nanogels (300 μg/mL) in serum-free medium at 37°C for 22 h, chloroquine (10 mg/mL) was added into every well for another 2 h to promote endosomal escape [22]. Then, Hela cells were washed with PBS and incubated in a nanogel-free medium and treated with an 808-nm laser at 400 mW/cm^2^ for 15 min and a 680-nm LED lamp at 10 mW/cm^2^ for 40 min. For cell survival test, the irradiated plates were returned to the incubator, and cell viability was colorimetrically measured 48 h later with MTT assay [23].

## Results and discussion

### Synthesis of Au_rod_@pNIPAAm-PEGMA nanogel

**Figure 1:**
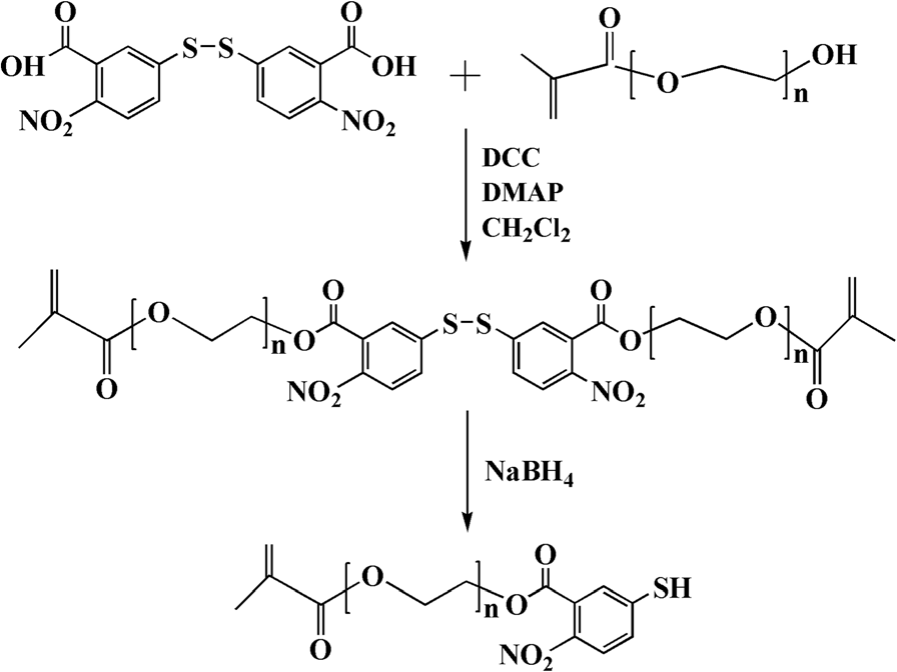
Schematic description of the synthesis of PEGMA-SH.

The synthesis of PEGMA-SH was shown in Figure 1. PEGMA-DTNB compound was firstly gained by the esterification reaction between the terminal hydroxyl group on the PEGMA and the carboxyl group on the DTNB with the DCC as medium and DMAP as catalyst [24,25]. Subsequently, the disulfide bond of PEGMA-DTNB was reduced by NaBH_4_ to yield the desired PEGMA-SH compound.

**Figure 2:**
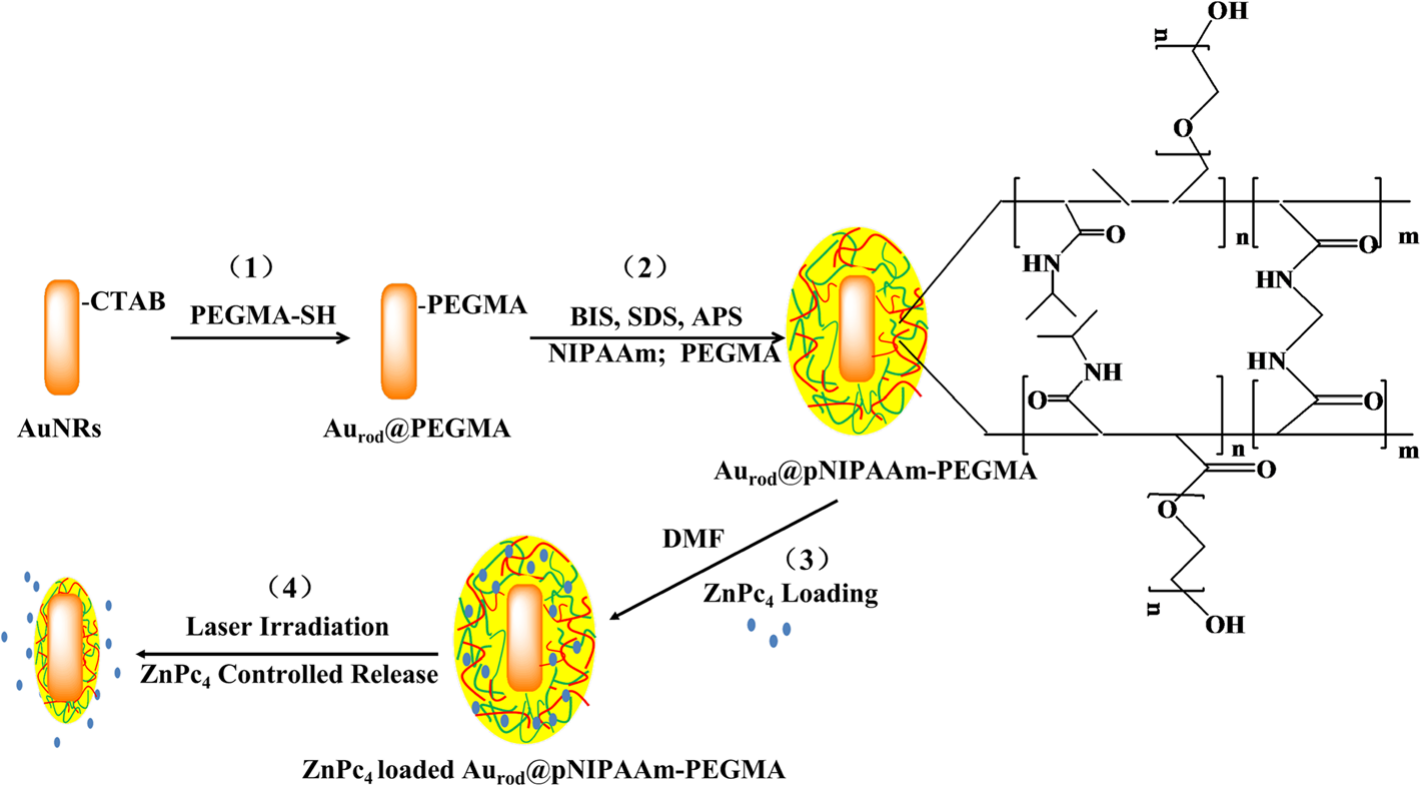
**Preparation of the Au**_**rod**_**@pNIPAAm-PEGMA nanogel.** (1, 2) Schematic of the sequence of steps in the synthesis of the hybrid Au_rod_@pNIPAAm-PEGMA nanogels, (3) ZnPc4 loading process, and (4) NIR-mediated ZnPc_4_ release.

**Figure 3:**
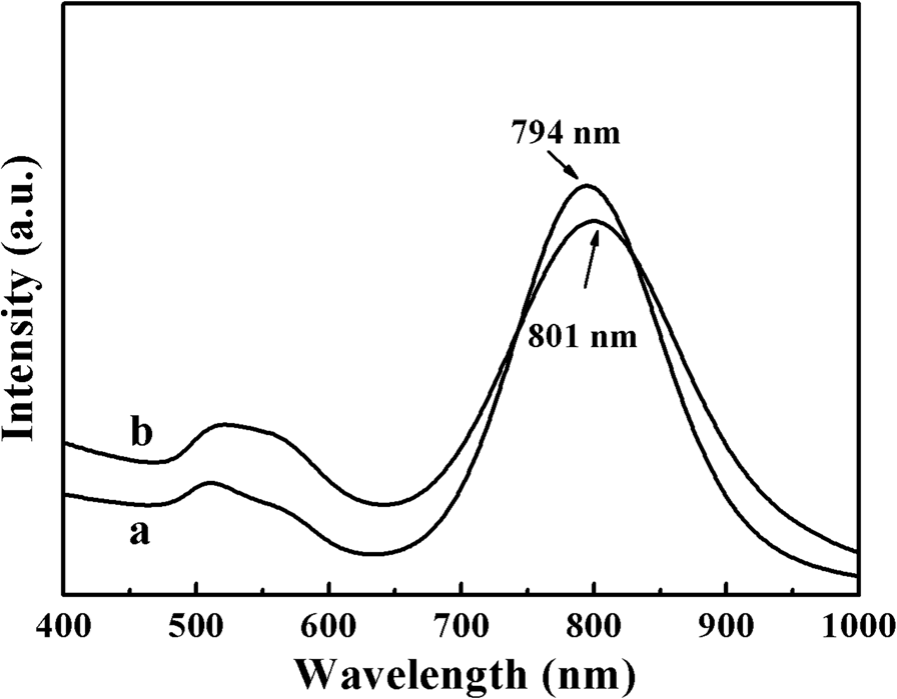
**The UV–vis spectra of (a) AuNRs and (b) Au**_**rod**_**@pNIPAAm-PEGMA nanogel.**

**Figure 4:**
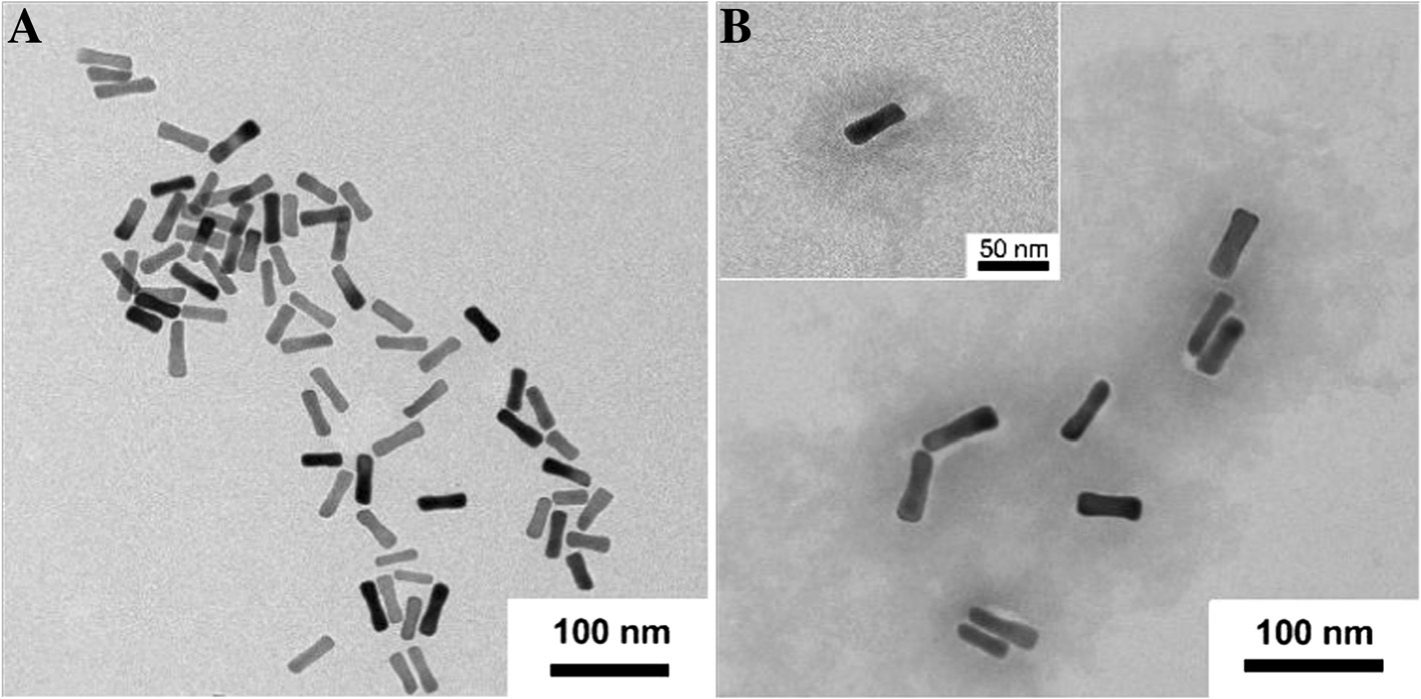
The typical TEM images of AuNRs (A) before and (B) after modification with pNIPAAM-PEGMA, respectively.

The strategy to prepare the Au_rod_@pNIPAAm-PEGMA nanogel involves two steps, growing a PEGMA monolayer on the surface of a AuNR, followed by *in situ* polymerization and cross-linking of NIPAAm and PEGMA, as depicted in Figure 2. In the first step, the AuNR surface was modified with a PEGMA self-assembled monolayer through a sulfhydryl-gold interaction. In the second step, PEGMA-modified AuNRs could be used as a template for *in situ* formation of hydrogel by polymerization and cross-linking of NIPAM and PEGMA with BIS as crosslinker, APS as initiator, and SDS as emulsifier. The coating of pNIPAAm-PEGMA on AuNRs can be reflected in the corresponding UV–vis spectra (Figure 3). AuNRs used in this work had a length of about 50 nm with an aspect ratio of approximately 3.2 (Figure 4A) which exhibited the maximum of the plasmon peak of 794 nm (Figure 3a). After the AuNRs were modified with pNIPAAm-PEGMA, a red shift from 794 to 801 nm occurred (Figure 3b). This red shift of SPR and the peak shape widening might be due to a change for AuNRs in the local refractive index produced by the pNIPAAm-PEGMA shell (Figure 4B) [26].

**Figure 5:**
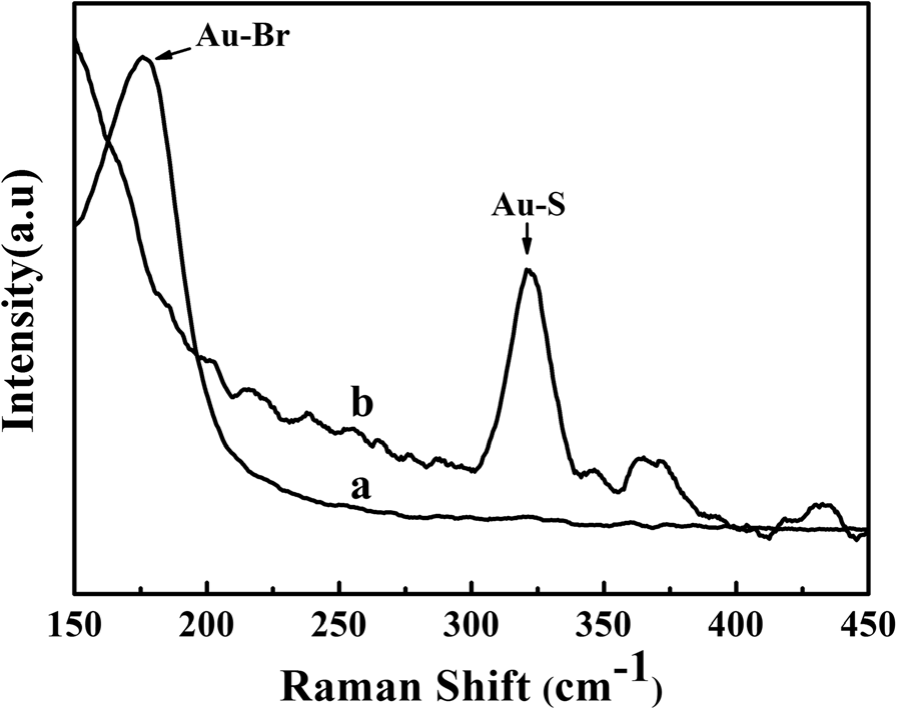
**The Raman spectra of (a) AuNRs and (b) Au**_**rod**_**@pNIPAAm-PEGMA nanogel.**

Raman spectra were also used to identify the synthesis of the Au_rod_@pNIPAAm-PEGMA nanogel. The Raman spectrum of the as-prepared AuNRs (Figure 5a) exhibited a band at 190 nm which was ascribable to the Au-Br bond on the surface of AuNRs [27]. This is because the as-prepared AuNRs were stabilized by the cationic detergent cetyltrimethylammonium bromide (CTAB) in the aqueous solution. After being modified with pNIPAAm-PEGMA (Figure 5b), the Au-Br band disappeared, and a band at 320 nm was observed, which was assigned to the Au-S bond [28]. It is thus suggested that PEGMA-SH might replace CTAB to form PEGMA-modified AuNRs through the Au-S bond, and then, PEGMA-SH on the surface of AuNRs might serve as the template for the following polymerization and cross-linking of NIPAAm and PEGMA.

**Figure 6:**
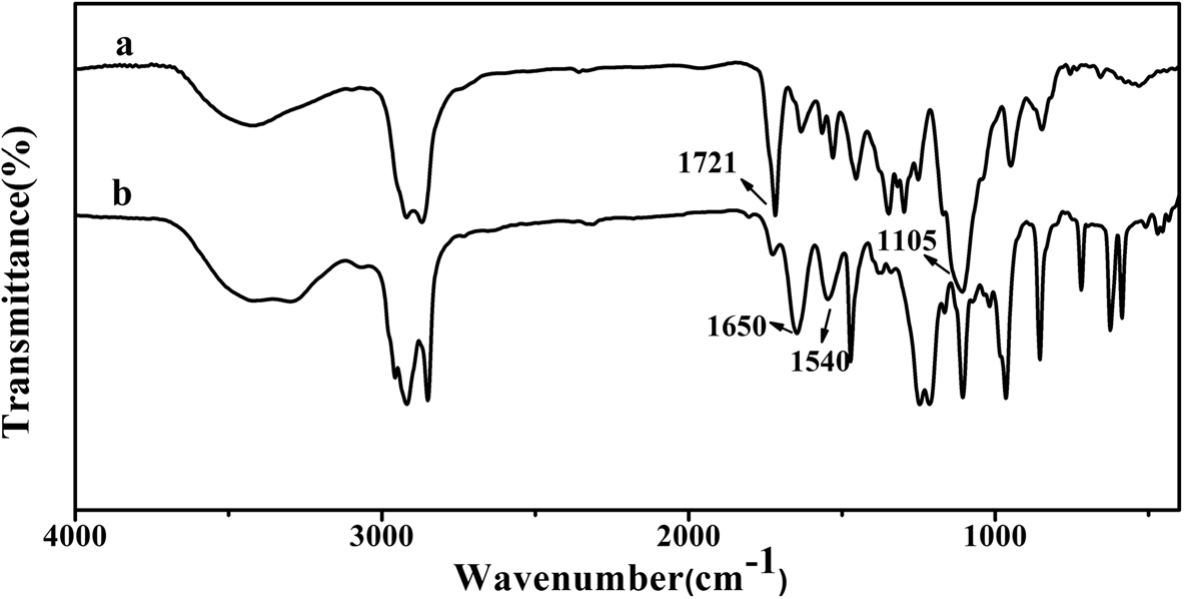
**FTIR spectra of (a) Au@PEGMA and (b) Au**_**rod**_**@pNIPAAm-PEGMA nanogel.**

FTIR spectra (Figure 6) were recorded to confirm the structure of the polymer shell. In the FTIR spectrum of PEGMA-modified AuNRs (Figure 6a), the absorption peaks of PEGMA, including *ν*(C=O) (1,721 cm^−1^) and *ν*(C-O-C) (1,105 cm^−1^), were observed. The spectrum of Au_rod_@pNIPAAm-PEGMA nanogels (Figure 6b) exhibited the characteristic peaks of polymerized NIPAAm at 1,650 cm^−1^ (*ν*(C=O), amide I) and 1,550 cm^−1^ (*δ*(N-H), amide II). Hence, the FTIR results could provide evidence for the surface modification and polymerization on AuNRs.

### Thermosensitive property of Au_rod_@pNIPAAm-PEGMA nanogel

**Figure 7:**
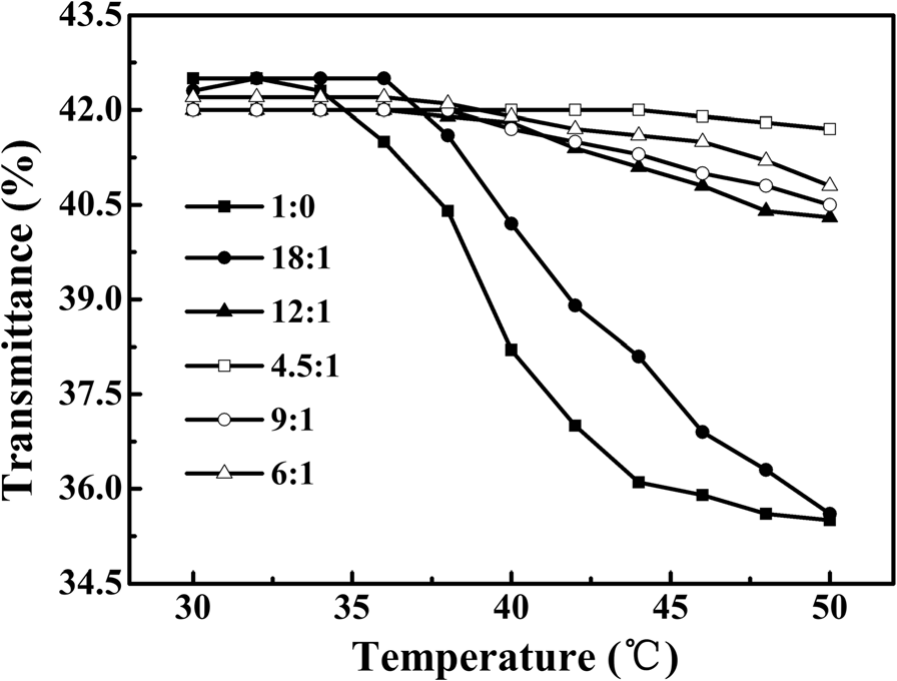
**The transmittance versus temperature curves of Au**_**rod**_**@pNIPAAm-PEGMA nanogels.** With different molar ratios of NIPAAm/PEGMA (1:0, 18:1, 12:1, 9:1, 6:1, 4.5:1, respectively).

Figure 7 and Table 1 showed the effect of the molar ratios of NIPAAm/PEGMA on the LCSTs of the Au_rod_@pNIPAAm-PEGMA nanogel. The Au_rod_@pNIPAAm (the molar ratio of NIPAAm/PEGMA, 1:0) exhibited an LCST of approximately 32°C, which was consistent with pure pNIPAAm [13]. It is clearly shown in Table 1 that the LCSTs of the Au_rod_@pNIPAAm-PEGMA nanogel could be tuned by changing the molar ratio of NIPAAm/PEGMA. Namely, as the molar ratio of NIPAAm/PEGMA decreased, the LCST of the nanogel increased. For example, when the molar ratio of NIPAAm/PEGMA was set at 18:1, the LCST of Au_rod_@pNIPAAm-PEGMA nanogels could be up to 36°C. The addition of hydrophilic PEGMA increased the hydrophilicity of pNIPAAm due to the strong interactions between water and hydrophilic groups on the polymer, which led to an increased LCST [29]. It is thus expected that this attractive property of tunable LCST might make Au_rod_@pNIPAAm-PEGMA nanogels more promising in drug delivery application.

**Table 1:** **The LCSTs of Au**_**rod**_**@pNIPAAm-PEGMA nanogels with different molar ratios of NIPAAm/PEGMA**

**NIPAAm (mmol)**	**PEGMA (mmol)**	**NIPAAm/PEGMA (mmol/mmol)**	**LCST (°C)**
1.8	0	1:0	32
1.8	0.1	18:1	36
1.8	0.15	12:1	38
1.8	0.2	9:1	40
1.8	0.3	6:1	42
1.8	0.4	4.5:1	44

### NIR-mediated ZnPc_4_ release

**Figure 8:**
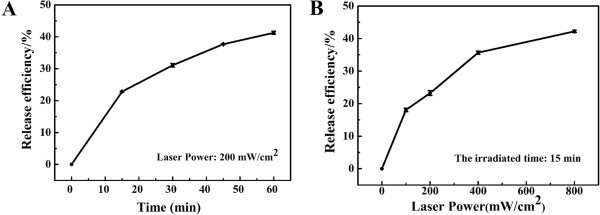
**NIR-mediated release of ZnPc**_**4**_**.** (**A**) Time- and (**B**) power-dependent of release of ZnPc_4_ from Au_rod_@pNIPAAm-PEGMA nanogels, respectively.

NIR-mediated release of ZnPc_4_ loaded in Aurod@pNIPAAm-PEGMA nanogels was investigated with the irradiation of a NIR laser (808 nm). When the sample was irradiated at 200 mW/cm^2^, the release efficiency was about 23.5% in the initial 20 min. As the irradiated time was prolonged, the cumulative release efficiency was up to 37.4% within 1 h (Figure 8A). This can be explained by the AuNRs of Au_rod_@pNIPAAm-PEGMA nanogels absorbing a certain SPR wavelength light and converting it into heat [30]. The heat diffused into the polymer shell and caused the shrinkage of the pNIPAAm-PEGMA nanogels and the release of ZnPc_4_.

The effect of laser power density on drug release was studied (Figure 8B). Exposure of Au_rod_@pNIPAAm-PEGMA nanogels to an 808-nm laser with the power of 100 mW/ cm^2^ for 15 min caused 20% of the loaded ZnPc_4_ released. More loaded ZnPc_4_ (43.7%) in Au_rod_@pNIPAAm-PEGMA nanogels could be released upon the irradiation power of 800 mW/ cm^2^. This is because when irradiated with a low-power NIR laser, small shrinkage of nanogels occurred, whereas a laser at high power might make nanogels shrink considerably and rapidly [31], consequently more ZnPc_4_ could be released. It is thus speculated that the NIR-responsive Au_rod_@pNIPAAm-PEGMA nanogel, acting as drug delivery carriers, could offer specific drug delivery to the targeted site, such as a tumor zone.

### Singlet oxygen detection

In PDT, the photosensitizing drugs should preferentially accumulate in target tissues and subsequently be activated by light with a matching wavelength to generate reactive singlet oxygen [32]. The singlet oxygen will cause the destruction of target cells by a complex cascade of chemical, biological, and physiological reactions [33]. The Au_rod_@pNIPAAm-PEGMA nanogels served as ZnPc_4_ carrier in PDT; besides the excellent properties of drug loading and release, its effect on the capability of loaded ZnPc_4_ to generate singlet oxygen was also investigated.

**Figure 9:**
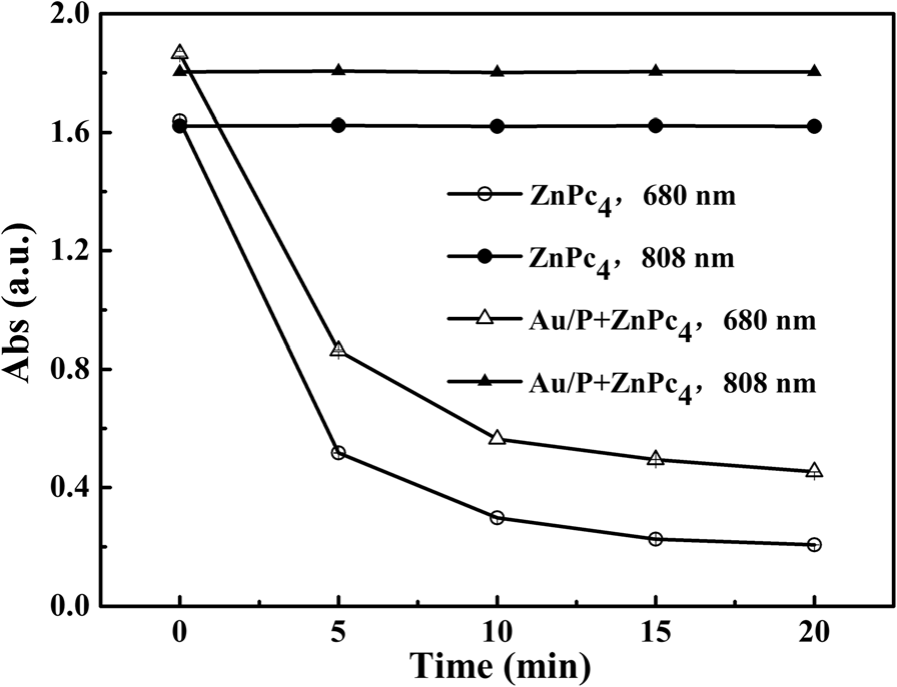
**The generation profiles of singlet oxygen from ZnPc**_**4**_**-loaded Au**_**rod**_**@pNIPAAm-PEGMA nanogels (Au/P).** The nanogels were irradiated by an 808-nm laser and a 680-nm LED lamp, respectively.

Photo-induced ^1^O_2_ of ZnPc_4_ was examined by a chemical method by using DMA, which could react with ^1^O_2_ to form an endoperoxide. The decrease in amount of DMA can be recorded by measuring the absorption at 377 nm. As shown in Figure 9, when ZnPc_4_-loaded Au_rod_@pNIPAAm-PEGMA nanogels or pure ZnPc_4_ was irradiated by an 808-nm laser, the absorption of DMA remained unchanged with the increase of exposure time to light, whereas the absorption of DMA continuously decreased as the ZnPc_4_-loaded Au_rod_@pNIPAAm-PEGMA nanogels or pure ZnPc_4_ was irradiated by a 680-nm light. This decrease indicated the production of ^1^O_2_, which can irreversibly react with DMA. Moreover, the generation curve of ZnPc_4_-loaded Au_rod_@pNIPAAm-PEGMA nanogels was similar with that of pure ZnPc_4_, demonstrating that the capacity of generating ^1^O_2_ of ZnPc_4_ was hardly affected after being loaded in Au_rod_@pNIPAAm-PEGMA nanogels. It is thus suggested that the Au_rod_@pNIPAAm-PEGMA nanogel might be a promising drug carrier for photodynamic therapy in the future.

### *In vitro* PDT study on Hela cells

**Figure 10:**
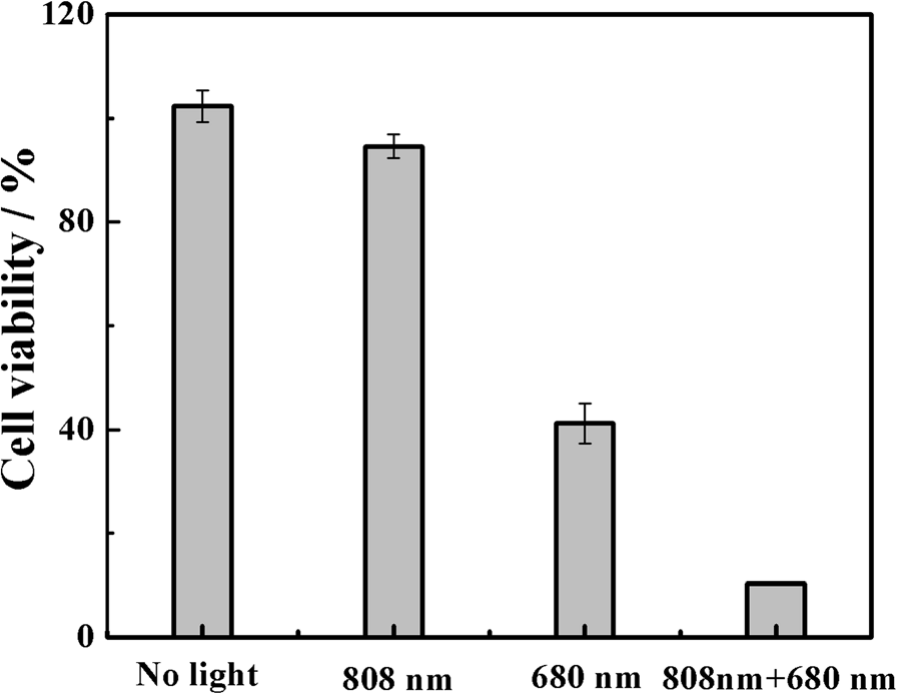
**The photodynamic therapy effect of ZnPc**_**4**_**-loaded Au**_**rod**_**@pNIPAAm-PEGMA nanogels on Hela cells at different irradiated conditions.**

The *in vitro* PDT study, represented in Figure 10, showed the percentage of cell viability after treatment of Hela cells with the ZnPc_4_-loaded Au_rod_@pNIPAAm-PEGMA nanogel (300 μg/mL) at different irradiated conditions. Compared with the cells’ group with no light treatment, no significant difference of the cell viability was found in the 808-nm laser-treated group. However, for the 680-nm light-treated group, the cell viability decreased to 40%. It is interesting to note that when irradiated by the two lights, the cell viability decreased to 10%. This is because the 808-nm laser treatment might result in the release of ZnPc_4_ from nanogels, which could improve the efficiency of the generation of singlet oxygen after the 680-nm irradiation and thus enhance the PDT effect on Hela cells.

## Conclusions

A facile approach to prepare near-infrared-responsive Au_rod_@pNIPAAm-PEGMA nanogels was described. The LCSTs of these Au_rod_@pNIPAAm-PEGMA nanogels could be tuned by changing the molar ratio of NIPAAm/PEGMA. The release of ZnPc_4_ loaded in Au_rod_@pNIPAAm-PEGMA nanogels increased with the extension of irradiated time and the increase of the power of the NIR laser. The loaded ZnPc_4_ in Au_rod_@pNIPAAm-PEGMA nanogels could generate singlet oxygen efficiently. The *in vitro* study showed obvious PDT effect on Hela cells. On these bases, the Au_rod_@pNIPAAm-PEGMA nanogels might serve as a promising drug carrier in PDT.

## Competing interests

The authors declare that they have no competing interests.

## Authors’ contributions

RL conceived the study, participated in the experimental design, and helped draft the manuscript. TXH participated in the design of the study and performed the statistical analysis. ST and WCD carried out the preparation experiments and drafted the manuscript. LDH, KXB, YAQ, and CM participated in the characterization experiments. All authors read and approved the final manuscript.

## Authors’ information

RL, TXH, and LDH are Ph.Ds. and professors. ST, WCD, KXB, YAQ, and CM are M.D. students in the Department of Biomaterials, College of Materials, Xiamen University.
